# Effectiveness and Safety of Shortened Postoperative Antibiotic Regimens in Children with Perforated Appendicitis: A Systematic Review and Meta-Analysis

**DOI:** 10.1055/a-2761-5649

**Published:** 2025-12-24

**Authors:** Pia Löfgren, Elias Berge, Elisabeth M.L. de Wijkerslooth, Lennart Jivegård, Sofia Sjöström, Susanna M. Wallerstedt

**Affiliations:** 1 Department of Pediatric Surgery, The Queen Silvia Children's Hospital, Sahlgrenska University Hospital, Gothenburg, Sweden; 2 Department of Radiology, Maasstad Hospital, Rotterdam, The Netherlands; 3 Center for Health Technology Assessment, Sahlgrenska University Hospital, Region Västra Götaland, Gothenburg, Sweden; 4 Department of Paediatrics, Sahlgrenska Academy, University of Gothenburg, Sweden; 5 Department of Pharmacology, Sahlgrenska Academy, University of Gothenburg, Gothenburg, Sweden

**Keywords:** antibiotic, children, meta-analysis, perforated appendicitis, systematic review

## Abstract

**Background:**

Postoperative antibiotic treatment of varying length is routinely used in perforated appendicitis to reduce the risk of complications. In the pediatric population, outcomes of shortened antibiotic regimens have not been specifically reviewed.

**Methods:**

Medline, Embase, the Cochrane Library, and CINAHL were searched (October 2023): P, patients: Children with perforated appendicitis; I, intervention and C, comparison: Antibiotic regimen below (I) and above (C) a defined number of days; O, outcomes, focus: Patient risks and benefits. For the main outcome (intra-abdominal abscess), non-inferiority was assessed.

**Results:**

Three randomized controlled trials (RCTs; 215 children) and one non-randomized study (288 children) fulfilled the PICO criteria. Regarding intra-abdominal abscess, pooling data from two RCTs (<5 vs. 5 days of intravenous antibiotics; 16 (17%) vs. 15 [15%] events) resulted in a wide 95% confidence interval (risk difference: −8 to 12 percentage points) not meeting the predefined non-inferiority margin of 7.5. One RCT (2 vs. 5 days of intravenous antibiotics) provided data regarding readmissions (9 vs. 7 events) and complications to antibiotic treatment (8 vs. 9 events). Two RCTs (<5 vs. 5 days of intravenous antibiotics) reported significantly shorter length of stay in the intervention group.

**Conclusion:**

This systematic review shows neither non-inferiority nor an increased risk of intra-abdominal abscess with a shortened postoperative antibiotic regimen. There may be no difference regarding readmission rates and treatment-related complications. Shorter regimens probably offer the advantage of reduced hospital stay. Due to substantial uncertainties, further RCTs are needed to define the optimal duration of antibiotics in children.

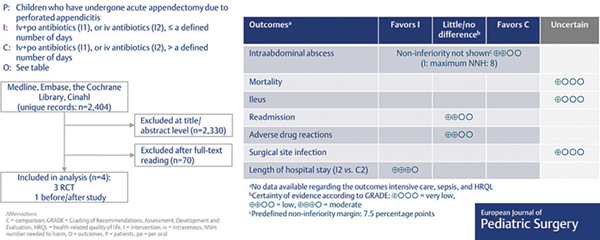

## Introduction

Acute appendicitis is a common cause for surgery in children worldwide. 1 Approximately 20% of these children have a perforated appendix, and the complication rate in this subgroup is 8% to 20%, mostly attributed to intra-abdominal abscess formation. 2 3 4 5 6 Intravenous antibiotics have historically been prescribed for up to 10 postoperative days or even longer to prevent severe complications. In recent years, however, there is a trend towards shorter treatment and a switch to oral antibiotics as soon as possible after surgery, an aspect that could be of importance for the length of stay, which may vary considerably. 7 8 Shortened antibiotic regimens—both the intravenous part and the entire course—would be preferable for both children and parents, provided they do not unacceptably increase risks, such as abscess rates. Earlier discharge would also be advantageous from a health care perspective since this could free up hospital beds to other children. Shortened antibiotic regimens could also be favorable in terms of antimicrobial resistance.

As far as we are aware, there are only two previous systematic reviews related to antibiotics in children after perforated appendectomy. 6 9 One concluded that sequential intravenous/oral antibiotic therapy is non-inferior to intravenous therapy only, based on meta-analyses that were not statistically significant, and the number of treatment days was not considered in the analyses. 6 The other focused on effects of the duration of postoperative antibiotic treatment for complex appendicitis, that is, gangrenous and/or perforated appendicitis with or without abscess formation, and no separate analyses for children were performed. 9

This systematic review was undertaken to investigate if a shortened postoperative regimen of intravenous/oral antibiotics is non-inferior to a regimen of more treatment days regarding the risk of intra-abdominal abscess, and whether it affects other important patient outcomes.

## Methods

This systematic review was primarily conducted within a health technology assessment (HTA), intended to provide information for regional evidence-based decision-making and including, for instance, the number of patients at issue in the region as well as regional economic aspects. 10 The systematic review was performed according to the established routines at the Center for Health Technology Assessment in Region Västra Götaland, Sweden, reported according to the Preferred Reporting for Systematic Reviews and Meta-Analyses (PRISMA) guidelines, 11 and preregistered with the International Prospective Register of Systematic Reviews (PROSPERO; registration number: CRD42024501215).

The aim was defined in the PICO format. Accordingly, patients (P) were children (0–17 years of age) who have undergone acute appendectomy due to perforated appendicitis, overall and in the subgroups with localized and diffuse peritonitis, respectively. The intervention (I) was a regimen of postoperative intravenous and oral antibiotics (I1), or intravenous antibiotics (I2), equal to or below a defined number of days. The comparison (C) was a regimen of postoperative intravenous and oral antibiotics (C1), or intravenous antibiotics (C2), more than a defined number of days. As the I and C were unspecific regarding the regimen treatment length in the comparison groups, all relevant literature, irrespective of the specific number of days of treatment, was captured. The main outcomes (O) were intra-abdominal abscess, mortality, ileus, need for intensive care, and sepsis. Additional outcomes were readmission, complication to antibiotic treatment, surgical site infection, length of stay, and health-related quality of life. As we aimed to analyze potential effects of a shortened regimen of antibiotics, publications were restricted to randomized controlled trials (RCTs) and prospective controlled studies. Languages were restricted to English, Swedish, Danish, or Norwegian. Beforehand, we defined three subgroups of interest: Children with preoperative vomiting, symptom duration before surgery ≤48 hours, and preoperative C-reactive protein >100. They all represent clinical features that may be of importance for the postoperative antibiotic regimen and the risk of complications.

### Literature Search and Study Selection

Two HTA librarians performed systematic searches in Medline, Embase, the Cochrane Library, and CINAHL (October 23, 2023). Reference lists of relevant articles were scrutinized for additional references. The websites of the Swedish Agency for Health Technology Assessment and Assessment of Social Services (SBU) and other local HTA centers were visited. To identify ongoing studies, a search in Clinicaltrials.gov was performed (February 21, 2024). Search strategies are provided in the Supplementary Material (available in the online version only).

The two librarians independently screened titles and abstracts to exclude publications that clearly did not meet the PICO, that is, the study selection criteria. Abstracts obtained in the first systematic search were screened using the Rayyan tool. 12 Discrepancies were resolved in consensus. For the remaining publications, the full texts were retrieved and were independently assessed by five authors (P.L., E.B., L.J., S.S., and S.M.W.) after which a consensus discussion took place among these authors to finally decide on inclusion or exclusion, that is, whether the PICO was fulfilled or not. No biases influenced the final decisions to include or exclude articles. For RCTs with mixed ages not reporting children separately, and RCTs focusing on complex appendicitis including gangrenous appendicitis but not reporting perforated appendicitis separately, we contacted the study investigators for additional details. For articles excluded in consensus, after full-text reading, reasons for exclusion were recorded.

### Data Extraction and Study Assessments

For included studies, data on design and methodology were extracted, as well as data regarding the participant characteristics and the antibiotic regimens of the intervention and the control groups. Number of outcome events or measures of effect were also extracted. Data were independently extracted by two authors, with discrepancies resolved in consensus.

At least two authors independently appraised the included studies using checklists used by Center for Health Technology Assessment, Region Västra Götaland, modified from checklists developed by the Swedish Agency for Health Technology Assessment and Assessment of Social Services. 13 These checklists contain three domains: Directness (external validity), risk of bias (internal validity), and precision. The directness domain includes questions regarding each component of the PICO, and the extent to which a study corresponds to this. The risk of bias domain is similar to the Cochrane Risk of Bias tool, 14 including four questions on potential biases related to the randomization process, the treatment, assessments, attrition, reporting, and conflict of interest. The third domain concerns sample size considerations and power. Consensus discussions were then performed among five authors (P.L., E.B., L.J., S.S., and S.M.W.) to decide on the domains' directness, study limitations (risk of bias), and precision, in the categories + (plus, no or minor problems); ? (question mark, some problems); and − (minus, major problems). The certainty of evidence was assessed using the Grading of Recommendations, Assessment, Development and Evaluation (GRADE) approach. 15 To communicate the findings, informative statements according to GRADE guidelines were used. 16

### Statistical Analysis

When two or more studies provided poolable data, we performed random effects meta-analyses using the software Review Manager (RevMan) version 5.4.1 (The Nordic Cochrane Centre, The Cochrane Collaboration, Copenhagen, Denmark) to obtain risk ratios (RRs) and risk differences (RDs), along with 95% confidence intervals (CIs). Before pooling, we determined whether the studies were poolable with focus on the compared treatment lengths of antibiotics. Meta-analyses based on studies without major risk of bias were beforehand determined to be the primary basis for the conclusions. Furthermore, we set the non-inferiority margin to 7.5 percentage points, based on the 95% CIs of the RDs, as this margin had been applied in a large and recent RCT where the authors considered the level justified under the assumption that infectious complications after appendicectomy for complex appendicitis would lead to minor morbidity. 17 In addition, the margin was discussed among the authors, including two pediatric surgeons (P.L. and S.S.), and found reasonable, in particular, as it would make the systematic reviews comparable. As a sensitivity analysis, we also applied a one-sided CI in the non-inferiority analysis; we considered it unlikely that a postoperative regimen of longer intravenous/oral antibiotic treatment would increase the risk of intra-abdominal abscess.

## Results

**Fig. 1:**
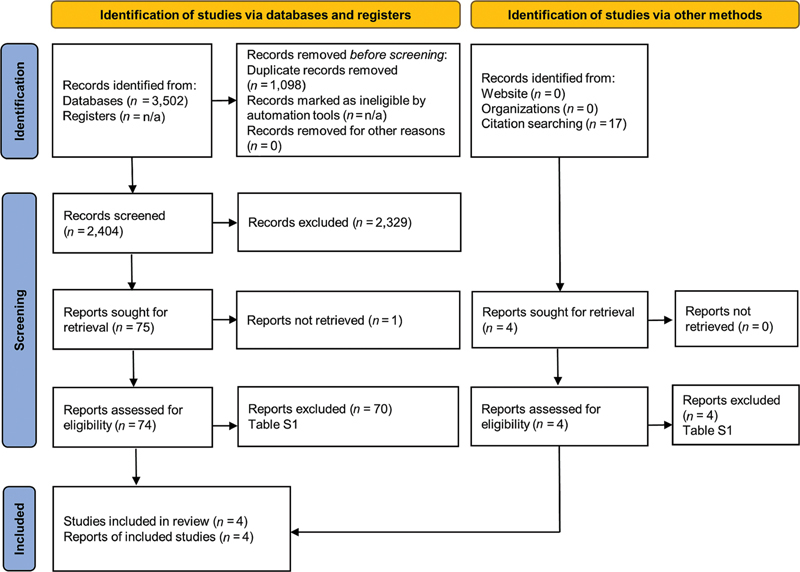
PRISMA flowchart. PRISMA, Preferred Reporting for Systematic Reviews and Meta-Analyses.

After removal of duplicates, the literature search identified 2,421 records, whereof two RCTs and one before/after study were directly included in this systematic review ( Fig. 1 ). 18 19 20 Another three RCTs reported results for a mixed population of children/adults and complex/perforated appendicitis combined. 4 17 21 Upon our request, separate data for children with perforated appendicitis were obtained from one of these, 17 and this RCT was consequently included in the analysis. For other RCTs, contact with the authors could not be established despite repeated attempts. 4 21 Consequently, they had to be excluded as they did not provide data that fulfilled our PICO. Publications excluded after full-text reading, as well as the reasons for excluding them, are presented in Supplementary Table S1 (available in the online version only).

Included studies are described in Table 1 , with further details provided in Supplementary Table S2 (available in the online version only). None of the RCTs was assessed to have major risk of bias ( Supplementary Table S3 [available in the online version only]). In Table 2 , we present a summary of our findings, including the certainty of evidence. No studies reported results regarding intensive care, sepsis, or health-related quality of life. No studies analyzed the predefined subgroups patients with localized or diffuse peritonitis separately, and further analyses of these subgroups were consequently not possible. Regarding the other predefined subgroups, we did not perform separate analyses as the data were restricted to those obtained upon request from one RCT 17 : No data were available with/without preoperative vomiting; three versus four events of intra-abdominal abscess occurred in children with symptom duration ≤48 hours; and five versus four such events occurred in those with a preoperative CRP >100.

**Table 1:** Characteristics of the included studies a

Study	Country	Study design	*n* (patients)	Antibiotic regimens compared	Main clinical findings
de Wijkerslooth et al, 2023 17 b	The Netherlands	RCT	87	2 versus 5 days IV	(I: 42 patients, C: 45 patients) • Intra-abdominal abscess: 6 versus 5 • Mortality: 0 versus 0 • Ileus: 1 versus 4 • Readmission: 9 versus 7 • Complication to antibiotic treatment: 8 versus 9 • Surgical site infection: 1 versus 0 • Length of stay (mean ± SD; days): 3.4 ± 1.6 versus 5.6 ± 1.9
Desai et al, 2015 18	United States	Before/After	288	<5 versus 7 days IV + oral	(I: 152 patients, C: 136 patients) • Intra-abdominal abscess: 12 versus 6
Fraser et al, 2010 19	United States	RCT	102	5 versus 7 days IV + oral; <5 versus 5 days IV	(I1: 52 patients, C1: 50 patients; I2: 50 patients, C2: 52 patients) • Intra-abdominal abscess: 10 versus 10 • Length of stay (mean ± SD; days): IV + oral: 6.06 ± 2.00 versus 4.48 ± 2.36 c IV: 4.48 ± 2.36 versus 6.06 ± 2.00 c *p* = 0.01
Rice et al, 2001 20	United States	RCT	26	<5 versus 10 days IV d	(I: 16 patients, C: 10 patients) • Intra-abdominal abscess: 0 versus 0 • Ileus: 0 versus 0 • Surgical site infection: 0 versus 1

Abbreviations: C, comparison; I, intervention; IV, intravenous; RCT, randomized controlled trial; SD, standard deviation.

Further details of the studies are provided in Supplementary Table S2 (available in the online version only).

a All studies included children with perforated appendicitis; definitions of perforation varied slightly.

b Data obtained from authors.

c sic erat scriptum, thus it was written.

d IV + oral: 10 treatment days were applied in both comparison groups.

**Table 2:** Summary of findings

Outcome	Number of studies	Number of patients in (pooled) analyses	Effect summary	Certainty of evidence (GRADE)
Intra-abdominal abscess a	3 RCTs, 1 before/after	189	Non-inferiority not shown; no statistically significant difference	Low b
Mortality	1 RCT	87	No difference	Very low c
Ileus	2 RCTs	113	No difference	Very low c
Readmission	1 RCT	87	No difference	Low b
Complication to antibiotic treatment	1 RCT	87	No difference	Low b
Surgical site infection	2 RCTs	113	No difference	Very low c
Length of stay	2 RCTs	189	Reduced by ∼2 days	Moderate d

Abbreviations: GRADE, Grading of Recommendations, Assessment, Development and Evaluation; NA, not applicable; RCT, randomized controlled trial.

a Non-inferiority approach applied for this outcome; predefined inferiority margin: 7.5 percentage points.

b Serious imprecision, some uncertainty regarding directness, some study limitations.

c Very serious imprecision, some uncertainty regarding directness, some study limitations.

d Some uncertainty regarding directness, some study limitations (no inconsistency and no imprecision as both studies showed similar and statistically significant results).

### Main Outcome: Intra-Abdominal Abscess

**Fig. 2:**
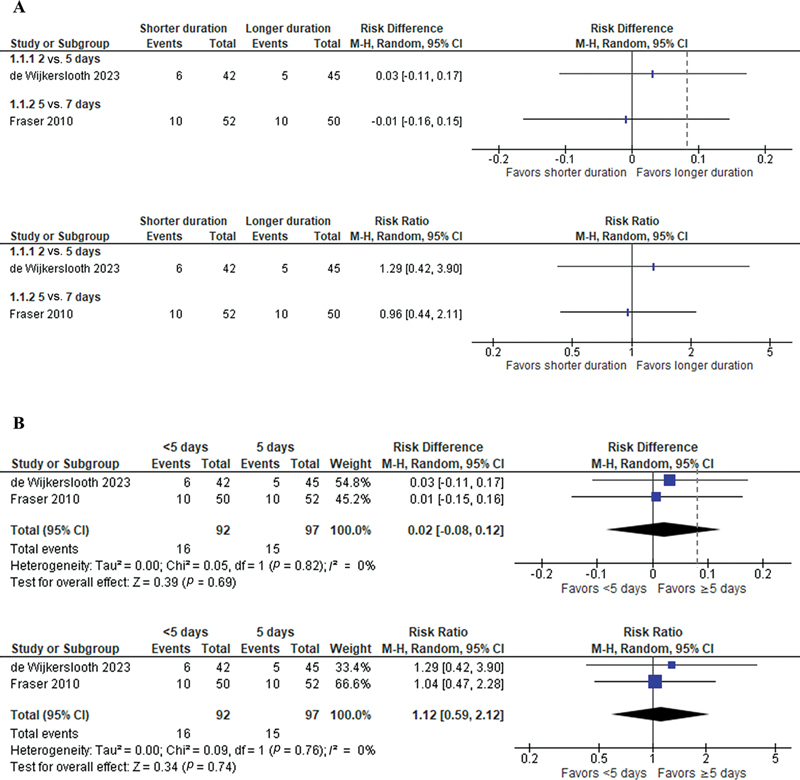
Forest plots of risk differences and risk ratios for intra-abdominal abscess in the two RCTs comparing two regimens regarding treatment lengths of postoperative antibiotics. ( **A** ) Intravenous plus oral; not poolable as a 5-day postoperative regimen of intravenous/oral antibiotic treatment was compared with a 7-day regimen in one study and with a 2-day regimen in the other. ( **B** ) Intravenous. The non-inferiority margin is presented with a dashed line. CI, confidence interval; RCT, randomized controlled trial.

Results regarding intra-abdominal abscess were available in all four studies, whereof two RCTs provided data regarding the entire antibiotics course. 17 19 One of the latter compared a 2- and a 5-day postoperative regimen of intravenous antibiotics not followed by oral treatment, 17 and the other compared 5- and 7-day regimens including both intravenous and oral antibiotics. 19 Consequently, pooling was not relevant, neither regarding the results nor the number of days applied in the comparison groups ( Fig. 2A ).

Regarding intravenous antibiotics alone, two RCTs could be pooled. 17 19 For one of these, the comparison groups were reversed regarding the intravenous part compared with the entire antibiotic course. 19 A total of 16 versus 15 events occurred in the randomization groups, resulting in a weighted RD of 2 percentage points (95% CI: −8 to 12, thus exceeding the non-inferiority margin of 7.5%; Fig. 2B ). In the sensitivity analysis where a one-sided CI was applied, the upper confidence limit was 11 percentage points. The RR was 1.12 (95% CI: 0.59–2.12).

### Other Outcomes

Regarding the outcomes mortality, ileus, and surgical site infections, there were too few events for conclusions.

Data on readmission were only available from one RCT comparing a 2- and a 5-day postoperative regimen of intravenous antibiotics not followed by oral treatment. 17 Nine (21%) versus seven (16%) readmissions occurred in the comparison groups resulting in an RR of 1.38 (95% CI: 0.56, 3.37) and an RD of 6 (95% CI: −10–22) percentage points.

Regarding complications to antibiotic treatment, eight versus nine events occurred in the comparison groups, resulting in an RR of 0.95 (95% CI: 0.41, 2.24) and an RD of −1 (95% CI: −18–16) percentage points.

Data on length of stay were available from two RCTs. 17 19 Regarding the entire antibiotics course, the RCTs could not be pooled as a 5-day postoperative regimen of intravenous/oral antibiotic treatment was compared with a 7-day regimen in one study 19 and with a 2-day regimen in the other. 17 Regarding intravenous treatment alone, the two RCTs, comparing <5 days and 5 days of postoperative antibiotics, could be pooled. Both studies reported statistically significant shorter length of stay in the intervention group. In one of the RCTs, the mean difference was −2.2 (95% CI: −2.94 to −1.46) days. 17 In the other, the difference could not be estimated based on reported figures. 19

The search in Clinicaltrials.gov resulted in 48 records. No additional studies fulfilled our PICO.

## Discussion

This systematic review shows that non-inferiority regarding the risk of intra-abdominal abscess cannot be established for a total antibiotics course shortened by 2 to 3 days in children with perforated appendicitis, or for an intravenous regimen of less than 5 days. On the other hand, an increased risk is not shown. Further, it remains uncertain whether shortened postoperative regimens of intravenous antibiotics affect mortality, ileus, and surgical site infections, primarily due to no or few events. Shortening the regimen of intravenous antibiotics by 3 days may not affect the readmission rate or the risk of complications from antibiotic treatment. Shortening the regimen of intravenous antibiotics, with a total treatment length of <5 days, probably reduces the length of hospital stay.

The predefined non-inferiority margin regarding intra-abdominal abscess was not met, and given the upper confidence limit of the absolute RD, that is, 12 percentage points, the worst-case scenario would be one extra event per eight children. Even if an abdominal abscess is a severe complication, it is clinically manageable within a reasonable time frame and has been described to lead to minor morbidity. 17 A reasonable clinical interpretation of these results—given that readmission rates may not be affected, and it is likely that the hospital stay can be shortened by approximately 2 days—this systematic review does not imply that shortened postoperative antibiotic regimens should be avoided.

Several recent studies in adults suggest algorithms for postoperative antibiotic treatment based on clinical criteria, including the ability to eat, normalization of white blood cell count, afebrile for >24 hours, and tolerable stomach pain. 22 23 24 25 26 Indeed, as the bioavailability of the antibiotics at issue is high, 27 a large fraction of oral formulations can be expected to reach the systemic circulation when the patient has started eating, has normalized bowel movement, and is not vomiting. Although our systematic review does not specifically address the switch from parenteral to oral administration, the pharmacokinetic characteristics of these drugs thus support switching to oral treatment based on clinical criteria, facilitating early discharge from the hospital.

For children operated for acute perforated appendicitis, the American Pediatric Surgical Association recommends intravenous broad-spectrum antibiotics for 5 days postoperatively, with a total treatment length of 7 days and allowing an early switch to oral antibiotics if allowed by clinical criteria. 28 Guidelines from the World Society of Emergency Surgery recommend a switch to oral antibiotics after 48 hours, with a total treatment time of less than 7 days. 29 However, no explicit link between the recommendations and the supporting evidence is provided in these guidelines, and the current evidence synthesis shows that the evidence underlying these recommendations in children is limited. Interestingly, recently updated guidelines, restricted to adults and referring to moderate-quality evidence, suggest limiting postoperative antibiotic treatment to 24 to 48 hours for patients with complicated appendicitis undergoing appendectomy, that is, those with gangrenous or perforated appendicitis. 30

An important strength of this systematic review is that it provides a synthesis of currently available evidence regarding patient-relevant outcomes for shortened antibiotic regimens after appendectomy in children with perforated appendicitis. Although the evidence was restricted, such a synthesis provides important information in health care as well as for future studies. Another strength is that our systematic review was guided by an established HTA process and included thorough and transparent assessments. A major limitation of the current review is that few studies fulfilled our PICO. In fact, no studies at all could be identified for some outcomes, and for others, the certainty of evidence was very low or inconclusive. In addition, it can be noted as a limitation that the youngest children were excluded from some studies. Another limitation concerns the meta-analyses. Indeed, our pooling of data was based on the intervention and control group as defined by the PICO. Thus, the antibiotic regimens and the treatment lengths varied within the pooled groups, and heterogeneity must be considered in the interpretation of the results. Further, although most appendectomies in the pooled studies were performed with a laparoscopic technique and the antibiotic substances were similar, it must be acknowledged that surgical technique, hospital protocols, and patient populations may vary between hospitals, 8 and may have implications for the results.

In conclusion, this systematic review highlights substantial uncertainties regarding the benefit–risk balance of shortened postoperative antibiotic regimens in children with perforated appendicitis. It does not, however, indicate that such regimens ought to be avoided. These findings encourage future research, preferably well-designed RCTs, to guide safe optimization of postoperative antibiotic strategies in children with perforated appendicitis, especially since this condition is relatively common.
