# Supplementary material for: Effectiveness and Safety of Shortened Postoperative Antibiotic Regimens in Children with Perforated Appendicitis: A Systematic Review and Meta-Analysis
Source: Eur J Pediatr Surg. 2025 Dec 24;36(3):210–6. doi: 10.1055/a-2761-5649 (PMC13288424; doi:10.1055/a-2761-5649)
Supplement: Supplementary file 1 — Supplementary Material [file 10-1055-a-2761-5649-s2024097082rev.pdf]

Thieme

Supplementary Material

Table of contents

Search strategies

Table S1	Studies excluded after full-text reading, and reasons for exclusion
Table S2	Detailed characteristics of the included studies
Table S3	Reasons underlying study assessments of directness and risk of bias

Search strategies

Database: Ovid MEDLINE(R) ALL
Date: 23 Oct 2023
No. of results: 1,114

#	Searches	Results
1	exp Appendicitis/	21258
2	exp Appendectomy/	12695
3	((appendicit* or appendix* or appendectom* or appendicectom*) adj3 (ruptur* or perforat* or complicated or complex)).ab,kf,ti.	4944
4	(surg* or operat* or postoperat* or perioperat* or intraoperative* or postsurg* or perisurg* or intrasurg* or appendectom* or appendicectom* or laparoscopic* or postlaparoscopic* or perilaparoscopic* or intralaparoscopic*).ab,kf,ti.	3542572
5	3 and 4	3746
6	1 or 2 or 5	27205
7	exp Child/	2165176
8	exp Adolescent/	2223262
9	exp Infant/	1257320
10	exp Pediatrics/	63145
11	(child* or adolesc* or teen* or preteen* or pre-teen* or juvenil* or youth* or preschool* or school* or kindergarten* or kinder-garten* or kid or kids or infant* or newborn* or new-born* or neonat* or neo-nat* or baby or babies or toddler* or minor or minors or pediat* or paediat* or immatur* or preterm* or pre-term*).ab,kf,ti.	3315131
12	7 or 8 or 9 or 10 or 11	5270828
13	exp Anti-Bacterial Agents/	821368
14	exp beta-Lactams/	139978
15	exp Aminoglycosides/	170799
16	exp Lincomycin/	8064
17	exp Nitroimidazoles/	20049
18	exp Sulfonamides/	133424
19	exp Trimethoprim/	12894
20	(antibiot* or anti-biot* or antimicrob* or anti-microb* or antibact* or anti-bact* or antimycobact* or anti-mycobact* or antimyco-bact* or anti-myco-bact* or bacteriocid* or bacterio-cid*).ab,kf,ti.	670318
21	13 or 14 or 15 or 16 or 17 or 18 or 19 or 20	1363793
22	6 and 12 and 21	1435
23	animals/ not (animals/ and humans/)	5126813
24	(animal or animals or rat or rats or mouse or mice or rodent or rodents or dog or dogs or cat or cats or hamster or hamsters or rabbit or rabbits or swine or murine or porcine or horses or horse).ti.	2117903
25	23 or 24	5545689
26	22 not 25	1433
27	(comment or editorial or letter).pt.	2198253
28	26 not 27	1391
**29**	**limit 28 to (danish or english or norwegian or swedish)**	**1114**

Database: Embase 1974 to 2023 October 20 (OvidSP)
Date: 23 Oct 2023
No. of results: 1,903

#	Searches	Results
1	exp acute appendicitis/	11363
2	exp appendectomy/	26576
3	((appendicit* or appendix* or appendectom* or appendicectom*) adj3 (ruptur* or perforat* or complicated or complex)).ab,kf,ti.	6114

European Journal of Pediatric Surgery | DOI 10.1055/a-2761-5649 | © The Author(s).

4	(surg* or operat* or postoperat* or perioperat* or intraoperative* or postsurg* or perisurg* or intrasurg* or appendectom* or appendicectom* or laparoscopic* or postlaparoscopic* or perilaparoscopic* or intralaparoscopic*).ab,kf,ti.	4580084
5	3 and 4	5019
6	1 or 2 or 5	33178
7	juvenile/	55743
8	exp adolescent/	1785852
9	exp child/	3109923
10	exp pediatrics/	128111
11	(child* or adolesc* or teen* or preteen* or pre-teen* or juvenil* or youth* or preschool* or school* or kindergarten* or kinder-garten* or kid or kids or infant* or newborn* or new-born* or neonat* or neo-nat* or baby or babies or toddler* or minor or minors or pediat* or paediat* or immatur* or preterm* or pre-term*).ab,kf,ti.	4104856
12	7 or 8 or 9 or 10 or 11	5565538
13	exp antibiotic agent/	1787631
14	exp sulfonamide/	404765
15	(antibiot* or anti-biot* or antimicrob* or anti-microb* or antibact* or anti-bact* or antimycobact* or anti-mycobact* or antimyco-bact* or anti-myco-bact* or bacteriocid* or bacterio-cid*).mp. [mp=title, abstract, heading word, drug trade name, original title, device manufacturer, drug manufacturer, device trade name, keyword heading word, floating subheading word, candidate term word]	1202847
16	13 or 14 or 15	2447508
17	6 and 12 and 16	2471
18	animal/ not (animal/ and human/)	1201231
19	(animal or animals or rat or rats or mouse or mice or rodent or rodents or dog or dogs or cat or cats or hamster or hamsters or rabbit or rabbits or swine or murine or porcine or horses or horse).ti.	2281306
20	18 or 19	3205600
21	17 not 20	2468
22	limit 21 to (article or article in press or conference paper or "review")	2058
**23**	**limit 22 to (danish or english or norwegian or swedish)**	**1903**

Database: The Cochrane Library
Date: 23 Oct 2023
No of results: 250 ref
Cochrane reviews: 2
Cochrane protocols: 0
Trials: 248
Editorials: 0
Special collections: 0
Clinical answers: 0

ID	Search	Hits
#1	MeSH descriptor: [Appendicitis] explode all trees	803
#2	MeSH descriptor: [Appendectomy] explode all trees	719
#3	((appendicit* OR appendix* OR appendectom* OR appendicectom*) NEAR/2 (ruptur* OR perforat* OR complicated OR complex)):ti,ab,kw (Word variations have been searched)	525
#4	(surg* OR operat* OR postoperat* OR perioperat* OR intraoperative* OR postsurg* OR perisurg* OR intrasurg* OR appendectom* OR appendicectom* OR laparoscopic* OR postlaparoscopic* OR perilaparoscopic* OR intralaparoscopic*):ti,ab,kw (Word variations have been searched)	384300
#5	#3 AND #4	486
#6	#1 OR #2 OR #5	1303
#7	MeSH descriptor: [Child] explode all trees	78615
#8	MeSH descriptor: [Adolescent] explode all trees	125922
#9	MeSH descriptor: [Infant] explode all trees	42054
#10	MeSH descriptor: [Pediatrics] explode all trees	1179
#11	(child* OR adolesc* OR teen* OR preteen* OR (pre NEXT teen*) OR juvenil* OR youth* OR preschool* OR school* OR kindergarten* OR (kinder NEXT garten*) OR kid OR kids OR infant* OR newborn* OR (new NEXT born*) OR neonat* OR (neo NEXT nat*) OR baby OR babies OR toddler* OR minor OR minors OR pediat* OR paediat* OR immatur* OR preterm* OR (pre NEXT term*)):ti,ab,kw (Word variations have been searched)	387925
#12	#7 OR #8 OR #9 OR #10 OR #11	387937
#13	MeSH descriptor: [Anti-Bacterial Agents] explode all trees	15279
#14	MeSH descriptor: [beta-Lactams] explode all trees	10565

#15	MeSH descriptor: [Aminoglycosides] explode all trees	10011
#16	MeSH descriptor: [Lincomycin] explode all trees	1013
#17	MeSH descriptor: [Nitroimidazoles] explode all trees	3043
#18	MeSH descriptor: [Sulfonamides] explode all trees	15475
#19	MeSH descriptor: [Trimethoprim] explode all trees	1390
#20	(antibiot* OR (anti NEXT biot*) OR antimicrob* OR (anti NEXT microb*) OR antibact* OR (anti NEXT bact*) OR antimycobact* OR (anti NEXT mycobact*) OR (antimyco NEXT bact*) OR (anti NEXT myco NEXT bact*) OR bacteriocid* OR (bacterio NEXT cid*)):ti,ab,kw (Word variations have been searched)	49874
#21	#13 OR #14 OR #15 OR #16 OR #17 OR #18 OR #19 OR #20	77018
#22	#6 AND #12 AND #21	307
#23	(clinicaltrials OR trialsearch):so	483266
#24	(conference proceeding):pt	226743
#25	#23 OR #24	710009
**#26**	**#22 NOT #25**	**250**

Database: CINAHL (EBSCOhost)
Date: 23 Oct 2023
No. of results: 235

#	Query	Results
**S22**	**S17 NOT S20**<br>Limiters - **Language: Danish, English, Norwegian, Swedish**	**235**
S21	S17 NOT S20	238
S20	S18 OR S19	190,427
S19	TI (animal OR animals OR rat OR rats OR mouse OR mice OR rodent OR rodents OR dog OR dogs OR cat OR cats OR hamster OR hamsters OR rabbit OR rabbits OR swine OR murine OR porcine OR horses or horse)	122,433
S18	(MH "animals") NOT ((MH "animals") AND (MH "human"))	88,841
S17	S6 AND S10 AND S16	238
S16	S11 OR S12 OR S13 OR S14 OR S15	146,455
S15	TI (antibiot* OR anti-biot* OR antimicrob* OR anti-microb* OR antibact* OR anti-bact* OR antimycobact* OR anti-mycobact* OR antimyco-bact* OR anti-myco-bact* OR bacteriocid* OR bacterio-cid*) OR AB (antibiot* OR anti-biot* OR antimicrob* OR anti-microb* OR antibact* OR anti-bact* OR antimycobact* OR anti-mycobact* OR antimyco-bact* OR anti-myco-bact* OR bacteriocid* OR bacterio-cid*)	85,603
S14	(MH "Trimethoprim+")	1,599
S13	(MH "Sulfonamides+")	9,744
S12	(MH "Metronidazole")	2,202
S11	(MH "Antibiotics+")	89,695
S10	S7 OR S8 OR S9	1,537,926
S9	TI (child* OR adolesc* OR teen* OR preteen* OR pre-teen* OR juvenil* OR youth* OR preschool* OR school* OR kindergarten* OR kinder-garten* OR kid OR kids OR infant* OR newborn* OR new-born* OR neonat* OR neo-nat* OR baby OR babies OR toddler* OR minor OR minors OR pediat* OR paediat* OR immatur* OR preterm* OR pre-term*) OR AB (child* OR adolesc* OR teen* OR preteen* OR pre-teen* OR juvenil* OR youth* OR preschool* OR school* OR kindergarten* OR kinder-garten* OR kid OR kids OR infant* OR newborn* OR new-born* OR neonat* OR neo-nat* OR baby OR babies OR toddler* OR minor OR minors OR pediat* OR paediat* OR immatur* OR preterm* OR pre-term*)	1,109,934
S8	(MH "Adolescence+")	604,463
S7	(MH "Child+")	755,271
S6	S1 OR S2 OR S5	5,109
S5	S3 AND S4	754
S4	TI (surg* OR operat* OR postoperat* OR perioperat* OR intraoperative* OR postsurg* OR perisurg* OR intrasurg* OR appendectom* OR appendicectom* OR laparoscopic* OR postlaparoscopic* OR perilaparoscopic* OR intralaparoscopic*) OR AB (surg* OR operat* OR postoperat* OR perioperat* OR intraoperative* OR postsurg* OR perisurg* OR intrasurg* OR appendectom* OR appendicectom* OR laparoscopic* OR postlaparoscopic* OR perilaparoscopic* OR intralaparoscopic*)	709,646
S3	TI ((appendicit* OR appendix* OR appendectom* OR appendicectom*) N2 (ruptur* OR perforat* OR complicated OR complex)) OR AB ((appendicit* OR appendix* OR appendectom* OR appendicectom*) N2 (ruptur* OR perforat* OR complicated OR complex))	1,011
S2	(MH "Appendectomy")	2,560
S1	(MH "Appendicitis")	4,040

The websites of **Statens beredning för medicinsk och social utvärdering (SBU)** , **Folkehelseinstituttet, a number of regional HTA units, International HTA Database** and **Nationellt kliniskt kunskapsstöd** were visited
23 Oct 2023
Nothing relevant to the question at issue was found

Source	Search terms / Browsing	No. of results	No. of relevant results
**SBU**<br>www.sbu.se<br>”Visa även träffar äldre än 5 år”	Blindtarm<br>Blindtarmsbihang<br>Blindtarmsinflammation<br>Appendix<br>Appendicit<br>Appendektomi<br>blindtarmsoperation	0<br>0<br>0<br>10<br>1<br>1<br>2	0<br>0<br>0<br>0<br>0<br>0<br>0
**Folkehelseinstituttet (Norge)**<br>https://www.fhi.no/ku/metodevurdering/	blindtarm<br>Blindtarmsvedhenget<br>blindtarmbetennelse<br>appendix<br>appendisitt<br>appendektomi<br>Blindtarmsoperasjon	1<br>0<br>0<br>0<br>0<br>0<br>0	0<br>0<br>0<br>0<br>0<br>0<br>0
**CAMTÖ**<br>https://www.regionorebrolan.se/sv/forskning/kontakt-och-organisation/hta-enheten-camto/	Browsat		0
**HTA Region Stockholm**<br>https://www.chis.regionstockholm.se/hta/rapporter/	Browsat		0
**Regional samverkansgrupp HTA (tidigare Metodrådet) i Sydöstra sjukvårdsregionen**<br>https://sydostrasjukvardsregionen.se/samverkansgrupper/hta/genomforda-bedomningar/	Browsat		0
**HTA Syd**<br>https://vardgivare.skane.se/kompetens-utveckling/sakkunniggrupper/hta-skane/#110365	Browsat		0
**Medicinska rådet, Region Dalarna**<br>https://www.regiondalarna.se/plus/vard/ovrig-halso--och-sjukvard/medicinska-radet/	Browsat		0
**International HTA Database** https://database.inahta.org/	(appendicitis or appendectomy or appendectomies or appendicectomy or appendicectomies)<br><br>Year: 2013 - 2023	7	0
**Nationellt kliniskt kunskapsstöd**<br>https://nationelltklinisktkunskapsstod.se	Blindtarm<br>blindtarmsbihang<br>blindtarmsinflammation<br>appendix<br>appendicit<br>appendektomi<br>blindtarmsoperation	0<br>0<br>0<br>11<br>10<br>0<br>0	0<br>0<br>0<br>0<br>0<br>0<br>0

Reference lists
A comprehensive review of reference lists brought 17 new records.

Ongoing trials

A search was performed in Clinicaltrials.gov (Februari 21st 2024) using the search terms *(appendicitis OR 4etronidazol OR appendix OR appendectomy OR appendectomies OR appendicectomy OR appendicectomies) AND (rupture OR ruptures OR ruptured OR perforation OR perforations OR perforated OR complicated OR complex) AND (surgery OR surgeries OR surgical OR surgically OR operative OR operatively OR operation OR operations OR operational OR operating OR postoperative OR postoperatively OR postoperation OR postoperational OR perioperative OR perioperatively OR perioperation OR perioperational OR 4etronidazole4 OR intraoperatively OR intraoperation OR intraoperational OR postsurgery OR postsurgeries OR postsurgical*

OR postsurgically OR perisurgery OR perisurgeries OR perisurgical OR perisurgically OR intrasurgery OR intrasurgeries OR intrasurgical OR intrasurgically OR appendectomy OR appendectomies OR appendectomized OR appendectomised OR appendicectomy OR appendicectomies OR appendicectomized OR appendicectomised OR laparoscopy OR laparoscopies laparoscopic OR laparoscopical OR laparoscopically OR postlaparoscopy OR laparoscopies OR postpostlaparoscopic OR postlaparoscopical OR postlaparoscopically OR perilaparoscopy OR perilaparoscopies OR perilaparoscopic OR perilaparoscopical OR perilaparoscopically OR intralaparoscopy OR intralaparoscopies OR intralaparoscopic OR intralaparoscopical OR intralaparoscopically) AND (child OR childs OR children OR childrens OR adolescent OR adolescents OR teen OR teens OR preteen OR preteens OR juvenile OR juveniles OR youth OR youths OR preschool OR preschools OR school OR schools OR kindergarten OR kindergartens OR kinder-garten OR kinder-gartens OR kid OR kids OR infant OR infants OR newborn OR newborns OR new-born OR new-borns OR neonate OR neonates OR neonatal OR neonatals OR neo-nate OR neo-nates OR neo-natal OR neo-natals OR baby OR babies OR toddler OR toddlers OR minor OR minors OR pediatric OR pediatrics OR paediatric OR paediatrics OR immature OR immatures OR immaturity OR immatureness OR immaturely OR preterm OR preterms OR pre-term OR pre-terms) AND (antibiotic OR antibiotics OR anti-biotic OR anti-biotic OR antimicrobial OR antimicrobials OR antimicrobic OR antimicrobics OR antimicrobe OR anti-microbial OR anti-microbials OR anti-microbic OR anti-microbics OR anti-microbe OR antibacterial OR antibacterials OR anti-bacterial OR anti-bacterials OR antimycobacterial OR antimycobacterial OR anti-mycobacterial OR anti-mycobacterial OR antimyco-bacterial OR antimyco-bacterial OR anti-myco-bacterial OR anti-myco-bacterial OR bacteriocidal OR bacteriocide OR bacteriocides OR bacterio-cidal OR bacterio-cide OR bacterio-cides). The search identified 48 trials. One of these represented an RCT already included in this systematic review (Fraser et al., 2010). No additional studies fulfilled our PICO.

European Journal of Pediatric Surgery | DOI 10.1055/a-2761-5649 | © The Author(s).

Table S1 Studies excluded after full-text reading, as well as the reason for excluding them.

Publication	Reason for exclusion
Abdulkareem et al. Surgical Research Communications 1992;12:337-41	Wrong population (non-perforated)
Anderson et al. Journal of the American College of Surgeons 2018;227:247-54	Wrong study design (retrospective)
Anonymous. British Journal of Surgery 1984;71:144-6	Mixed population (ages). Wrong comparison (different drugs)
Anonymous. Emergency Medicine (00136654) 1992;24:200-3	Wrong comparison (different drugs)
Basoli et al. Journal of Gastrointestinal Surgery 2008;12:592-600	Wrong population (adults)
Berne et al. Surgery, Gynecology and Obstetrics 1993;177:18-22; discussion 35-40	Wrong population (adults)
Bueno-Rodríguez et al. Cirugía y Cirujanos 2012;80:233-8	Wrong language (Spanish)
Bufo et al. Pediatric Endosurgery and Innovative Techniques 1997;1:127-30	Wrong intervention/comparison (acute appendectomy vs antibiotics and delayed appendectomy)
Cho et al. International Journal of Surgery (London, England) 2016;27:142-6	Mixed population (ages). Non-RCT
Cunningham et al. Journal of Pediatric Surgery 2020;55:1026-31	Wrong intervention (pathway of antibiotic regimen, number of days not specified)
Di Benedetto et al. Journal of Chemotherapy 1989;1:814-5	Wrong study design (case series)
el-Mufti et al. Chemotherapy 1989;35:383-8	Mixed population (ages). Wrong I/C (ceftriaxone versus metronidazole/gentamicin/ampicillin)
Evbuomwan et al. East African Medical Journal 1994;71:279-81	Wrong focus (clinical classification, no comparison of antibiotic duration)
Fink et al. Ugeskrift for Laeger 1979;141:366-8	Mixed population (ages). Wrong I/C (cefaloridin vs penicillin/streptomycin)
Fishman et al. Journal of Pediatric Surgery 2000;35:923-6	Wrong comparison (hospital vs at home)
Flannigan et al. Surgery, Gynecology and Obstetrics 1983;156:209-11	Wrong intervention (no postoperative intravenous antibiotics)
Gollin et al. American Surgeon 2002;68:1072-4	Wrong study design (case series)
Gottrup et al. Ugeskrift for Laeger 1979;141:2293-6	Wrong comparison (different drugs)
Higginbotham et al. Nursing Case Management 1998;3:26-35	Wrong focus (development and implementation of pathway, control unclear)
Höllwarth et al. Zeitschrift fur Kinderchirurgie[Surgery in infancy and childhood] 1986;41:14-8	Wrong language (German)
Irfan Farooqi et al. Pakistan Journal of Medical and Health Sciences 2021;15:1248-50	Mixed population (ages). Wrong I (only preoperative antibiotics)
Jewett et al. Journal of Pediatric Surgery 1971;6:121-3	Wrong comparison (sulfadiazine as add on vs no sulfadiazine, duration unclear in control group)
Kekomaki et al. Zeitschrift für Kinderchirurgie 1981;32:310-4	Wrong comparison (different drugs)
Khan et al. Journal of Surgical Research 2020;256:390-6	Wrong comparison (before vs after protocol)
Kling et al. Acta Chirurgica Scandinavica 1985;151:73-6	Wrong comparison (different drugs)
Kooi et al. Clinical Therapeutics 1990;12:54-60	Wrong comparison (different drugs)
Lansdale et al. Journal of Pediatric Surgery 2019;54:272-5	Wrong population (complicated appendicitis including gangrenous appendicitis). Non-RCT
Lindahl. Ugeskrift for Laeger 1969;131:1637-40	Mixed population (ages). Non-RCT
Liu et al. Surgical Infections 2020;21:778-83	Wrong population (including gangrenous appendicitis). Data matching the PICO requested but not obtained.
Lose et al. Annales Chirurgiae et Gynaecologiae 1986;75:270-3	Mixed population (ages). Non-RCT
Maltezou et al. European Journal of Clinical Microbiology and Infectious Diseases 2001;20:643-6	Wrong comparison (different drugs)
Mazuski et al. Surgical Infections 2017;18:1-76	Wrong design (guidelines)
Meller et al. Surgery 1991;110:764-7; discussion 7-8	Wrong comparison (different drugs)
Miholic et al. Zeitschrift für Kinderchirurgie 1983;38:159-62	Wrong comparison (different drugs and administrations)
Naess. Tidsskrift for den Norske Laegeforening 1968;88:284-5	Wrong publication type (summary)
Ong et al. Singapore Medical Journal 2008;49:615-8	Wrong study design (retrospective)

Perez et al. International Journal of Infectious Diseases 2011;15:e569-75	Wrong comparison (different doses)
Pinto et al. British Medical Journal 1980;280:275-7	Mixed population (ages). Wrong I/C (metronidazol vs ampicillin/no antibiotic)
Pogorelic et al. Surgical Infections 2019;20:625-30	Wrong comparison (different drugs)
Pokorny et al. Surgery, Gynecology and Obstetrics 1991;172:54-6	Wrong comparison (different drugs)
Puri et al. Zeitschrift für Kinderchirurgie 1981;32:111-5	Wrong comparison (different drugs)
Raahave et al. Archives of Surgery 1986;121:924-9	Wrong comparison (part 1: no comparison, part two: placebo)
Raahave et al. Acta Chirurgica Scandinavica 1970;136:715-23	Wrong comparison (different drugs)
Raffensperger et al. Chicago Medical School Quarterly 1969;28:179-83	Wrong comparison (different drugs)
Rangel et al. Annals of Surgery 2017;266:361-8	Wrong study design (retrospective)
Robinson et al. Annals of Surgery 2017;266:195-200	Wrong comparison (before vs after guideline implementation)
Russell et al. Journal of Pediatric Surgery 2023;26:26	Wrong intervention (number of days with intravenous antibiotics not specified)
Samelson et al. Archives of Surgery 1987;122:691-6	Wrong intervention (protocol, no comparison of duration of antibiotics between groups)
Sawyer et al. New England Journal of Medicine 2015;372:1996-2005	Wrong P (intraabdominal infection). Wrong I/C (total number of days not specified)
Schmitt et al. Pediatrie 1993;48:633-7	Wrong comparison (different drugs)
Schmitt et al. Journal of Antimicrobial Chemotherapy 1989;24:157-63	Wrong language (French)
Schultz et al. Ugeskrift for Laeger 1979;141:363-5	Mixed population (ages). Wrong I/C (minocyclin vs penicillin/streptomycin)
Shandling et al. Journal of Pediatric Surgery 1974;9:79-83	Wrong study design (retrospective)
Shbat et al. Journal of Pediatric Surgery 2014;49:1723-5	Wrong population (non-perforated)
Sirinek et al. Clinical Therapeutics 1987;9:420-8	Mixed population (ages). Wrong I/C (cefoxitin vs clindamycin/gentamicin)
Sirinek et al. Surgery, Gynecology and Obstetrics 1991;172:30-5	Wrong comparison (different drugs)
Skarda et al. Journal of Pediatric Surgery 2014;49:1726-9	Wrong intervention (number of days with intravenous antibiotics not specified)
Slusher et al. Journal of Pediatric Surgery 2014;49:1020-4; discussion 4-5	Wrong intervention/comparison (different surgical strategies)
Solomkin et al. Annals of Surgery 2003;237:235-45	Wrong population (adults)
Sorooshian et al. Annals of the Royal College of Surgeons of England 2022;104:210-5	Wrong study design (retrospective)
St Peter et al. Journal of Pediatric Surgery 2008;43:981-5	Wrong comparison (different drugs)
Stone. American Family Physician 1971;4:60-8	Wrong study design (case series)
Stovroff et al. Journal of Pediatric Surgery 1994;29:245-7	Wrong intervention/comparison (with vs without peripheral inserted central line)
Svensson. Journal of Pediatrics 2016;176:221-4	Wrong publication type (comment)
Talei et al. Iranian Journal of Medical Sciences 1994;19:95-100	Mixed population (ages). Wrong I/C (metronidazol versus penicillin/chloramphenicol/gentamicin)
Taylor et al. American Surgeon 2004;70:858-62	Mixed population (ages). Wrong I/C (total number of days not specified)
Teppler et al. Journal of Chemotherapy 2004;16:62-9	Wrong population (adults)
Theodorou et al. Journal of Surgical Research 2022;275:48-55	Wrong study design (retrospective)
van den Boom et al. Digestive Surgery 2020;37:101-10	Wrong design (systematic review)
van Rossem et al. JAMA Surgery 2016;151:323-9	Mixed population (ages). Non-RCT
Vennits et al. Ugeskrift for Laeger 1990;152:157-60	Mixed population (ages, diagnosis). Data matching the PICO requested but not obtained.
Wang et al. BMC Pediatrics 2019;19:407	Wrong design (systematic review)
Willis et al. Pediatric Infectious Disease Journal 2018;37:429-35	Wrong intervention (antimicrobial stewardship, practice guideline)
Yu et al. European Journal of Pediatric Surgery 2014;24:341-9	Wrong population (including gangrenous appendicitis). Non-RCT

C = comparison, I = intervention, P = Population, RCT = randomized controlled trial

Table S2 Detailed characteristics of the included studies

**Publication**<br>**Country**	**Study design**<br>**Surgery**	**Patients (n)**<br><br>**Definition of perforated appendicitis**<br><br>***Exclusion criteria***	**Age, years (mean±SD)**<br><br>***Inclusion criteria***	**Female sex (%)**	**Subgroups**[1]	**Intervention**	**Comparison**
de Wijkerslooth et al. Lancet 2023;401:366-76<br>NL<br>(15 hospitals)	RCT<br><br>Laparoscopic surgery: 95%	I: 42<br>C: 45<br><br>Intraoperative perforation or abscess (gangrenous appendicitis in the absence of perforation or abscess excluded)<br><br>*Exclusion criteria: pregnancy, immuno-compromised, allergy to antibiotic agent, inadequate source control during surgery*	I1/I2: 12.7±3.1<br>C1/C2: 13.1±3.0<br><br>*Inclusion criteria: 8–17 years*	I1/I2: 29%<br>C1/C2: 29%	Preoperative vomiting NR<br><br>Symptom duration before surgery ≤48 hours<br>I1/I2: 57%<br>C1/C2: 87%<br>(P=0.002)<br><br>Preoperative serum CRP >100<br>I1/I2: 40%<br>C1/C2: 38%	<u>I1/I2:</u><br>IV: cefuroxine (1500 mg x3) or ceftriazone (2000 mg x1) +<br>metronidazole (500 mg x3)<br>**2 days**<br><br>The dose was adjusted according to weight.<br><br><u>Deviations from the allocated treatment:</u><br>• If intraoperative culture results necessitated another antibiotic regimen<br>• If early discontinuation was justified because of adverse effects or IV administration failure<br>• If postoperative infectious complications occurred	<u>C1/C2:</u><br>IV: cefuroxine (1500 mg x3) or ceftriazone (2000 mg x1) +<br>metronidazole (500 mg x3)<br>**5 days**
Desai et al. J Pediatr Surg 2015;50:912-4<br>US	Cohort (Before/ after)<br><br>Laparoscopic surgery: 100%	I: 152<br>C: 136<br><br>Hole in the appendix or fecalith in the abdomen<br><br>*Exclusion criteria: NR*	NR<br><br>*Inclusion criteria: NR*	NR	NR	<u>I1:</u><br>IV: ceftriaxone (50 mg/kg) + metronidazole (30 mg/kg) <5 days<br>If normal WBC count: No oral antibiotic;<br>**total: <5 days** of antibiotic treatment<br><br>(If elevated WBC count; amoxicillin-clavulanate ≥2 days, total: 7 days of antibiotic treatment)	<u>C1:</u><br>IV: ceftriaxone (dose NR) + metronidazole (dose NR) <5 days<br>Oral: amoxicillin-clavulanate ≥2 days,<br>**total: 7 days** of antibiotic treatment

European Journal of Pediatric Surgery | DOI 10.1055/a-2761-5649 | © The Author(s).

						Deviations from the allocated treatment:<br>• If discharge criteria (regular diet, oral analgesics, afebrile ≥12h) were not met and WBC count was elevated, IV antibiotics were administered for 2 more days	
Fraser et al. J Pediatr Surg 2010;45:1198-202<br>US	RCT<br><br>Laparoscopic surgery: 100%	I1/C2: 52<br>C1/I2: 50<br><br>Hole in the appendix or fecalith in the abdomen. Pre-operatively known intra-abdominal abscess<br><br>*Exclusion criteria: known severe concomitant process, pre-operatively known intra-abdominal abscess*	I1/C2: 9.7±4.2<br>C1/I2: 10.1±4<br><br>*Inclusion criteria: up to 18 years*	I1/C2: 40%<br>C1/I2: 40%	NR	I1:<br>IV: ceftriaxone + metronidazole (dose NR) 5 days<br>If normal WBC count: No oral antibiotic; **total: 5 days** of antibiotic treatment<br><br>(If elevated WBC count: prolonged IV antibiotics ≥2 days)	C1:<br>IV: ceftriaxone + metronidazole (dose NR) until tolerating diet<br>If <5 days: ≥2 days with oral amoxicillin-clavulanate (dose NR), **total: 7 days** of antibiotic treatment
						I2 (=C1 above):<br>IV: ceftriaxone + metronidazole (dose NR) until tolerating diet<br>If **<5 days**: ≥2 days with oral amoxicillin-clavulanate (dose NR), total: 7 days of antibiotic treatment	C2 (=I1 above):<br>IV: ceftriaxone + metronidazole (dose NR) **5 days**<br>If normal WBC count: No oral antibiotic<br><br>(If elevated WBC count: prolonged IV antibiotics ≥2 days)
						Deviations from the allocated treatment:<br>• If hospitalised with fever and elevated WBC count ≥day 7, or if returning to hospital after discharge with abdominal pain and elevated WBC count, a CT scan was performed and if an abscess was found, IV antibiotics were administered for 2 weeks.	

Rice et al. Arch Surg 2001;136:1391-5<br>US	RCT<br><br>Open surgery: 100%	I: 16<br>C: 10<br><br>Obvious perforation of the appendix accompanied by presence of purulence<br><br>*Exclusion criteria: pregnancy, renal failure, neutropenia, drug allergy, laparoscopic appendectomy*	I: 11.9±3.9<br>C: 12.5±3.7<br><br>*Inclusion criteria: 5-18 years*	I: 5<br>C: 4	NR	I2:<br>IV: ampicillin (400 mg/kg x4) + gentamicin (6.5 mg/kg x3) + clindamycin (40 mg/kg x3); until return of GI function; **maximum 5 days**<br><br>(PO: amoxicillin-clavulanate (40 mg/kg x3) + metronidazole (40 mg/kg x3) Total antibiotic duration: 10 days)	C2:<br>IV: ampicillin (400 mg/kg x4) + gentamicin (6.5 mg/kg x3) + clindamycin (40 mg/kg x3); **10 days**
						Deviations from the allocated treatment:<br>• If additional antibiotics was needed as determined by the physician	

C = comparison, CT = computer tomography, GI = gastrointestinal, I = intervention, IV = intravenous, NA = not applicable, NL = the Netherlands, NR = not reported, RCT = randomized controlled trial, SD = standard deviation, US = United States, WBC = white blood cell

[1]Predefined subgroups were with/without preoperative vomiting, symptom duration before surgery ≤/48 hours, and preoperative CRP >100

[2]Data obtained from authors

European Journal of Pediatric Surgery | DOI 10.1055/a-2761-5649 | © The Author(s).

Table S3 Aspects regarding directness, study limitations, and precision identified during the assessment process contributing to the study being categorised as having no/minor (+), some (?) or major (-) problems. These assessments applied to all outcomes if not explicitly stated otherwise.

**Publication Country**	**Study design**	**Problems contributing to downgrading the study in the assessment**					
		**Directness**		**Study limitations**		**Precision**	
de Wijkerslooth et al. Lancet 2023;401:366-76 NL (15 hospitals)	RCT	?	Children <8 years of age not included. In the entire study, not restricted to age, 27% of eligible patients (1,041/3,880) were excluded for unclear reasons.	?	Not blinded assessments. Symptom duration before surgery was more often ≤48 hours in the control group (87% *vs.* 57%). Protocol violation not evenly distributed between randomization groups.	+	
Desai et al. J Pediatr Surg 2015;50:912-4 US	Before/after	+		?/-	Confounding factors sparsely reported (e.g. not duration of symptoms and CRP). Adherence to protocol NR. Not blinded assessments.	-	Power calculations NR. Non-significant results interpreted as non-inferiority.
Fraser et al. J Pediatr Surg 2010;45:1198-202 US	RCT	?	Participant flowchart NR	?	Adherence to protocol NR. Not blinded assessments.	-	Power calculation performed, study preterminated before calculated sample size was recruited despite non-significant results in interim analysis.
Rice et al. Arch Surg 2001;136:1391-5 US	RCT	-	Children <5 years not included. Reasons for declined participation NR. Length of antibiotic treatment in C out of date (10 days). Open surgery.	?	Length of intravenous treatment not clearly predefined in I and C. Not blinded assessments.	-	Power calculation NR, pilot study. Non-significant results interpreted as non-inferiority.

C = comparison, NL = the Netherlands, NR = not reported, RCT = randomized controlled trial, US = United States

European Journal of Pediatric Surgery | DOI 10.1055/a-2761-5649 | © The Author(s).
